# Preparation and Characterization of an Injectable and Photo-Responsive Chitosan Methacrylate/Graphene Oxide Hydrogel: Potential Applications in Bone Tissue Adhesion and Repair

**DOI:** 10.3390/polym14010126

**Published:** 2021-12-30

**Authors:** Daniela N. Céspedes-Valenzuela, Santiago Sánchez-Rentería, Javier Cifuentes, Mónica Gantiva-Diaz, Julian A. Serna, Luis H. Reyes, Carlos Ostos, Christian Cifuentes-De la Portilla, Carolina Muñoz-Camargo, Juan C. Cruz

**Affiliations:** 1 Grupo de Investigación en Nanobiomateriales, Ingeniería Celular y Bioimpresión (GINIB), Department of Biomedical Engineering, Universidad de los Andes, Bogota 111711, Colombia; dn.cespedes@uniandes.edu.co (D.N.C.-V.); s.sanchezr2@uniandes.edu.co (S.S.-R.); jf.cifuentes10@uniandes.edu.co (J.C.); mr.gantiva@uniandes.edu.co (M.G.-D.); ja.serna10@uniandes.edu.co (J.A.S.); 2 Grupo de Investigación en Biomecánica (IBIOMECH), Department of Biomedical Engineering, Universidad de los Andes, Bogota 111711, Colombia; cc.cifuentes@uniandes.edu.co; 3 Department of Chemical and Food Engineering, School of Engineering, Universidad de Los Andes, Carrera 1 No. 18A-12, Bogota 111711, Colombia; lh.reyes@uniandes.edu.co; 4 Grupo CATALAD, Instituto de Química, Universidad de Antioquia, Medellin 050010, Colombia; carlos.ostos@udea.edu.co

**Keywords:** bioadhesive, chitosan methacrylate, graphene oxide, bone repair, photocrosslinking

## Abstract

As life expectancy continues to increase, the inevitable weakening and rupture of bone tissue have grown as concerns in the medical community, thus leading to the need for adhesive materials suitable for bone repair applications. However, current commercially available adhesives face certain drawbacks that prevent proper tissue repair, such as low biocompatibility, poor adhesion to wet surfaces, and the need for high polymerization temperatures. This work aims to develop an injectable and photo-responsive chitosan methacrylate/graphene oxide (ChiMA/GO) adhesive nanocomposite hydrogel of high biocompatibility that is easy to apply by simple extrusion and that offers the possibility for in situ polymer and physiological temperatures. The nanocomposite was thoroughly characterized spectroscopically, microscopically, rheologically, thermally, and through mechanical, textural, and biological assays to fully evaluate its correct synthesis and functionalization and its performance under physiological conditions that mimic those observed in vivo. In addition, a finite element analysis (FEA) simulation was used to evaluate its performance in femur fractures. Results suggest the material’s potential as a bioadhesive, as it can polymerize at room temperature, shows superior stability in physiological media, and is capable of withstanding loads from body weight and movement. Moreover, the material showed remarkable biocompatibility as evidenced by low hemolytic and intermediate platelet aggregation tendencies, and high cytocompatibility when in contact with osteoblasts. The comprehensive studies presented here strongly suggest that the developed hydrogels are promising alternatives to conventional bone adhesives that might be further tested in vivo in the near future.

## 1. Introduction

Comminuted fractures are complex bone lesions that involve the break or splinter of a bone into multiple fragments, usually after being subjected to loads far superior to those it can resist [1]. These injuries require a surgical treatment to allow the bone to properly heal and regain its ability to sustain strain, which is generally based on the reduction of the fractured surfaces, coupling of processed fragments, and stabilization by fixation implants or adhesive materials [2,3,4,5,6]. The main disadvantage of internal fixation devices (e.g., screws, plates, and wires) is the need for a second procedure to remove non-resorbable materials after the initial intervention, which usually results in additional complications such as refractures, infections, and other wound healing disorders [7,8,9]. In contrast, adhesive materials, which bond fragments together through surface adhesion and internal cohesion, are intended to be replaced gradually by re-growing bone, thus preventing the need for additional interventions. Moreover, these adhesives enable uniform distribution of physical forces over the bonded area, avoiding physical stress and tissue damage during the fixation and repair processes [10,11,12]. Over the past two decades, engineered biomaterials have been tailored to yield desirable biocompatibility and reduced foreign body responses, therefore emerging as promising next-generation adhesives for biomedical applications [11,12].

Misnomered as an adhesive, polymethyl methacrylate (PMMA) or bone cement is considered the gold standard for implant fixation in orthopedic surgery. Because it lacks intrinsic adhesive properties, bone cement relies on the close mechanical interlock between irregular surfaces to transfer forces from bone-to-bone or bone-to-prosthesis [13,14,15]. However, PMMA also shows low adhesion on wet surfaces, which makes it largely unsuitable for living bone tissue applications. Additionally, it induces persistent inflammation, which is undesirable for bone regeneration, and demands high polymerization temperatures (66–80 °C), a condition that might lead to tissue necrosis [13,14,15,16]. Attempts for overcoming these limitations have focused mainly on adding excipients such as powdered bioactive compounds to enhance biocompatibility and consequently accelerate the fixation process. This occurs at the expense of sacrificing partially its mechanical and handling properties [16,17,18,19]. A next generation of clinically usable bone adhesives must therefore comply with a few requirements, including (1) being easily sterilizable and biocompatible, (2) being capable of properly stabilizing the bone fragments for at least two weeks, (3) avoiding major interference with the bone healing processes, (4) being easy to apply, and (5) degrading without generating toxic metabolites [11,12,20].

Hydrogels might be an alternative to tackle the challenges presented above. These polymers are hydrophilic, three-dimensional networks capable of absorbing, retaining, and releasing water under different physical and chemical stimuli. Either synthetic or biobased, hydrogels have emerged as powerful platforms for tissue engineering and drug delivery [21,22]. Depending on the type of monomers, pendant chains, and degree of crosslinking, hydrogels can undergo reversible sol–gel phase transitions (and consequently changes in their properties) in response to specific stimuli such as temperature, pH, and light [21,23]. For example, several authors have reported tuning the mechanical properties of hydrogels through photo-crosslinking for 3D bioprinting and in situ deposition applications [24,25]. Additionally, hydrogels have proven suitable for wound healing as they are non-irritant, permeable to metabolites, and provide a moist and cell-friendly environment [26,27]. This healing potential has been combined with the remarkable adhesiveness of various biobased polymers to develop therapeutic wound dressings and bioadhesives for skin, intestine, and bone applications [26,27,28,29,30,31,32,33].

One of the most employed natural polymers for therapeutic hydrogels is chitosan, a biomaterial that can be obtained from the partial deacetylation of naturally abundant chitin [34,35,36,37]. Along with a strong inherent adhesiveness and biocompatibility, chitosan exhibits desirable hemostatic, antimicrobial, and analgesic properties, making it an attractive material for numerous applications in nanomedicine [38,39] and regenerative medicine [40]. Moreover, chitosan can be further engineered for specific functionalities by conjugating functional molecules to the free acetamide and hydroxyl groups linked to the glucopyranose rings in their structure, which are susceptible to nucleophilic attacks [37]. For instance, the conjugation of methacrylic acid (or methacrylation) has been used to enhance the adhesive properties of chitosan for dental applications [41]. Complementarily, graphene oxide (GO), a two-dimensional nanomaterial obtained from the exfoliation-oxidation of graphite, has been incorporated into chitosan hydrogels and scaffolds to form nanocomposites that further improve mechanical properties [42,43,44]. GO is biocompatible, biodegradable, antibacterial, physiologically stable, and sensitive to temperature and pH changes to elicit drug release, but several reports have also discussed its promising potential as reinforcement to enhance mechanical response and colloidal stability, mimic tissue microenvironments, and promote bone regeneration [45,46,47,48]. Moreover, the abundance of oxygen-rich functional groups on its surface makes it suitable for relatively simple chemical functionalization schemes and protein adsorption [42,43,44,49,50]. Nevertheless, recent reports have also shown that GO can bioaccumulate and induce inflammatory and cytotoxic responses, and thus only low doses are recommended for biological applications.

This work was dedicated to embedding GO in a covalently photocrosslinked network of chitosan methacrylate (ChiMA) to develop a biobased adhesive nanocomposite hydrogel that promotes bonding of fractured bone tissue under normal physiological conditions. Extensive physicochemical and biological characterization tests were carried out on the synthesized material to evaluate comprehensively its potential as a bioadhesive, including mechanical response, swelling and degradation, microscopic imaging, methacrylation level, and biocompatibility. Moreover, we conducted in silico analyses of the mechanical performance to gain a more detailed mechanistic understanding of the bioadhesive response under different load and compression conditions. The material put forward here holds much promise as a platform that can be tailored for matching the mechanical properties of different tissues through light-directed polymerization, and that is suitable for in situ deposition through extrusion-based methods.

## 2. Materials and Methods

### 2.1. Materials

High-density chitosan, methacrylic acid (MA), N-[3-(dimethylamino)-propyl]-N’-ethyl carbodiimide hydrochloride (EDC), N-hydroxysulfosuccinimide (NHS), dimethylformamide (DMF), riboflavin (RF), phosphate-buffered saline (PBS), 3-(4,5-Dimethyl-2-thiazolyl)-2,5-diphenyl-2H-tetrazolium bromide (MTT), and triton were purchased from Sigma-Aldrich (St. Louis, MO, USA). High-glucose Dulbecco’s modified Eagle medium (DMEM) was purchased from Gibco (Amarillo, TX, USA). Hydrogen peroxide (H_2_O_2_), sodium hydroxide (NaOH), glacial acetic acid, sulfuric acid, phosphoric acid, potassium permanganate (K_2_MnO_4_), hydrochloric acid (HCl), and Tris-HCl were purchased from PanReac AppliChem (Chicago, IL, USA). A commercial lactate dehydrogenase (LDH) cytotoxicity assay kit was purchased from Novus Biologicals (Littleton, CO, USA). Fetal bovine serum (FBS) was purchased from BioWest (Riverside, MO, USA), and graphite flakes were purchased from Graphene Supermarket (Ronkonkoma, NY, USA). The *Cercopithecus aethiops* kidney (Vero) cell line (ATCC^®^ CCL-81) was obtained from the American Type Culture Collection (Manassas, VA, USA). The Normal Human Osteoblast (NHOst) cell line (CC-2538) and Osteoblast Growth and Differentiation Basal Medium (OBM) were purchased from Lonza Bioscience (Basel, Switzerland). Polyester fiber and bovine femur samples were purchased from local shops.

### 2.2. Chitosan Methacrylation

**Figure 1:**
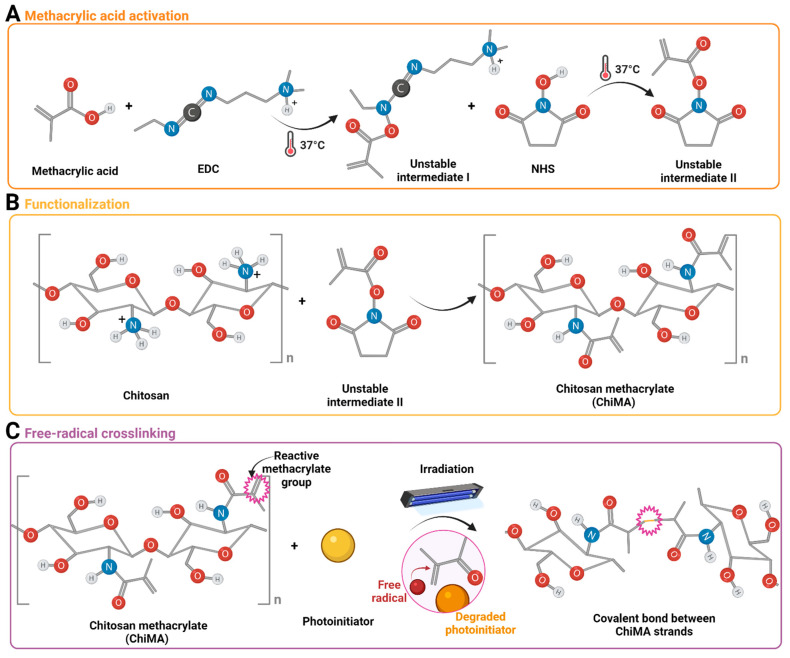
(**A**) Activation of methacrylic acid mediated by EDC and NHS. (**B**) Chemical conjugation of methacryloyl groups to the free amines of glucosamine units in chitosan monomers. (**C**) Methacryloyl group destabilization and crosslinking induced by the light-directed degradation of a photoinitiator (riboflavin).

Methacryloyl groups from MA were covalently bound to the pendant primary amines of chitosan monomers through EDC/NHS-mediated activation, following a modification of the protocol put forward by Shen et al. (Figure 1) [51]. High-density chitosan was solubilized in acetic acid 0.17 M at 3.5 mg/mL for 10 min under magnetic stirring. Furthermore, MA, EDC, and NHS were mixed in 3 mL of DMF 99.8% (*v*/*v*) at 1:1, 1:2, and 1:4 molar ratios with respect to the estimated free-amine groups in chitosan and activated for 15 min at 37 °C before their addition to the chitosan solution. These mixtures were then left to react for 24 h at 60 °C under continuous magnetic stirring at 600 RPM. The synthesized ChiMA formulations were dialyzed for 48 h against acetic acid 0.17 M and lyophilized for 48 h before sterilization with ethylene oxide.

The conjugation of methacryloyl groups to chitosan was evaluated by Fourier-transform infrared spectroscopy (FTIR) using an A250 FTIR (Bruker, Germany) instrument in the spectral range of 4000–400 cm^−1^. Chemical modifications were also assessed by X-ray Photoelectron Spectroscopy (XPS) using a NAP-XPS spectrometer (SPECS Surface Nano Analysis GmbH) equipped with a PHOIBOS 150-1D-DLD analyzer and a monochromatic Al-Kα source (1486.7 eV, 13 kV, 100 W). The survey spectra were obtained after five cycles using a pass energy of 100 eV and an energy step of 1 eV. For high-resolution spectra, the setup was fixed at 20 cycles, 20 eV pass, and 0.1 eV energy steps. An electron flood gun at 3 eV and 20 µA was employed for charge compensation. The C1s at 284.6 eV were selected for binding energy calibration. The fitting was performed via a Shirley-type background and optimized in the least-square method under well-established conditions of 30 GL ratio (Gaussian–Lorentzian peak shape), fullwidth half-maximum (FWHM), and peak position from the literature. A semi-quantification approach for the methacrylate functional groups was conducted from the decomposition C1s sub-peak components associated with the CO– and COO– species and N1s sub-peak component associated with the –O–N–C species. The atomic ratio composition was performed based on corrected peak areas for each spectrum and the pristine chitosan sample as a reference.

### 2.3. Synthesis of Graphene Oxide

**Figure 2:**
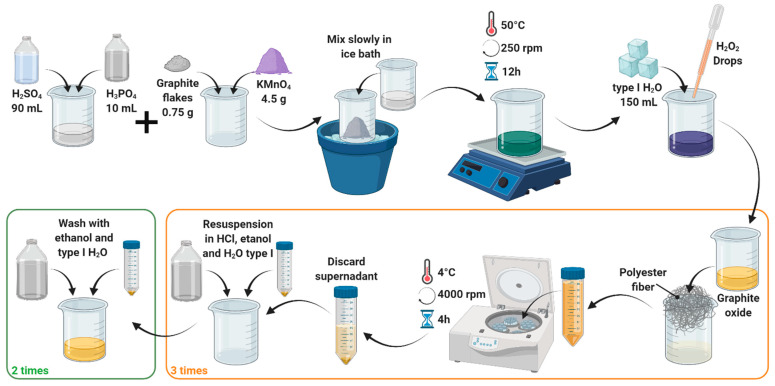
Synthesis of graphene oxide by the coupled exfoliation/oxidation of graphite, as described by Marcano’s modification of the Tour’s method.

GO was synthesized by graphite’s coupled exfoliation/oxidation, following Tour’s method (Figure 2) [52]. Briefly, a mixture of 90 mL of sulfuric acid 98% (*v*/*v*) and 10 mL of phosphoric acid 85% (*v*/*v*) was slowly added to 0.75 g of graphite flakes and 4.5 g of potassium permanganate and left to react under constant magnetic stirring at 50 °C. After 12 h, 150 mL of type I water ice cubes and 3 mL of hydrogen peroxide 30% (*w*/*w*) were added to the viscous solution, thus inducing a color change from dark purple to gold yellow. The obtained GO was sonicated for 5 min in an ultrasonic bath at an amplitude of 38% and a frequency of 40 kHz and washed successively aided by filtration with polyester fiber. Each washing cycle involved centrifugation at 4000 RPM for 4 h followed by resuspension in a washing solution containing 50 mL of HCl 30% (*v*/*v*), 50 mL of ethanol 96% (*v*/*v*), and 50 mL of type I water. The final pellet was resuspended in type I water and lyophilized for 24 h.

The proper synthesis of GO was confirmed through FTIR, Raman spectroscopy, thermogravimetric analysis (TGA), and transmission electron microscopy (TEM). FTIR spectra were recorded with an A250 FTIR (Bruker, Germany) instrument in the spectral range of 4000–400 cm^−1^, while Raman spectra were recorded with an XploRA Confocal Raman Microscope (Horiba Scientific, Japan). TGA profiles were collected with a TG analyzer (TA Instruments, New Castle, DE, USA) using a temperature ramp of 25–600 °C at a heating rate of 10 °C/min under a nitrogen atmosphere, and TEM imaging was conducted at 15,000× with a Tecnai F30 Microscope (Fei Company, Hillsboro, OR, USA).

### 2.4. Preparation of the Bioadhesive

**Figure 3:**
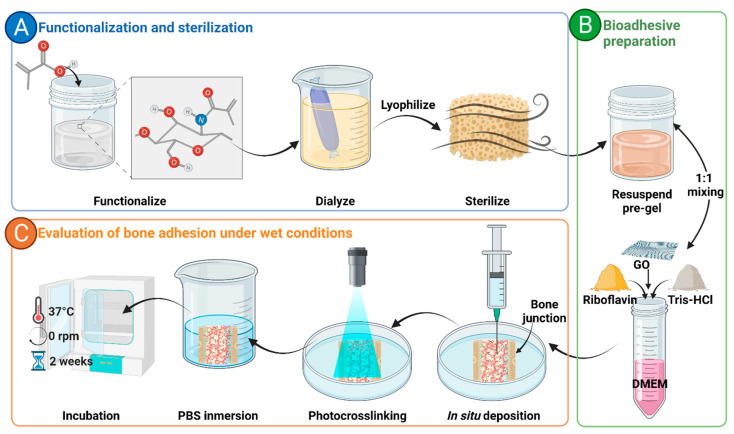
General methodology employed for the development of the bioadhesive. (**A**) Functionalization and sterilization of chitosan methacrylate (ChiMA). (**B**) Preparation of the bioadhesive with graphene oxide (GO) and riboflavin. (**C**) Evaluation of the adhesive potential under wet conditions.

Lyophilized ChiMA samples were resuspended at 40 mg/mL and 60 mg/mL in acetic acid 0.02 M. In parallel, a working solution of DMEM supplemented with 10% (*v*/*v*) FBS, 0.1% (*w*/*v*) RF, and Tris-HCl 0.1 M was prepared and adjusted to a pH of 8.5 by dripping NaOH 5M slowly. Resuspended ChiMA was mixed at a 1:1 volume ratio with the working solution, thus accounting for the GO-free formulations (ChiMA2% and ChiMA3%). For a GO-laden hydrogel, the nanomaterial was dispersed at 1 mg/mL in the working solution before forming the mixture (ChiMA3%GO) [53]. The overall process is shown in Figure 3.

Hydrogels were loaded into 3 mL syringes and manually ejected through a 21-gauge needle to assess their potential to form continuous filaments during extrusion. After deposition, samples were irradiated for 10 min at 62 mW/cm^2^ with blue light (420–460 nm) to induce covalent crosslinking of methacryloyl groups in the main backbone of ChiMA [24,53].

### 2.5. Rheological Evaluation

Experiments to evaluate the rheological behavior of bioadhesive samples were performed in a Discovery Series Hybrid Rheometer-1 (TA Instruments, New Castle, DE, USA) using a parallel plate geometry with a 20 mm gap. Changes in both the storage (G′) and loss (G′′) moduli of photocrosslinked samples were assessed through flow, frequency, time, and temperature sweeps. The flow sweep experiments were conducted from 0.01 to 100 Hz at 1% strain and room temperature. Frequency sweep experiments were performed between 0.01 and 100 rad/s at room temperature. Last, coupled time/temperature sweep experiments were carried under oscillatory mode, with a constant strain of 1% and 10 rad/s with a temperature ramp of 20 °C/min between 15 °C and 37 °C. Shear-thinning properties of the hydrogels were estimated by fitting the viscosity (*η)* vs. shear rate (*γ*) data to the power law model (Equation (1)):

(1) $$\eta =K\gamma^{n-1}$$

### 2.6. GO Dispersion

The nanomaterial’s self-fluorescence at 405 nm was imaged through confocal microscopy to evaluate GO dispersion within the hydrogel. ChiMA3%GO samples were imaged at 20× with an FV1000 Confocal Microscope (Olympus, Tokyo, Japan), and particle count and area were then analyzed aided by ImageJ^®^ software. The spatial distribution of GO sheets within the nanocomposite hydrogel was determined by a Z-stack reconstruction of images captured at different depth positions [53].

### 2.7. Morphological Analysis of Hydrogels

The polymeric microstructure of the hydrogels was imaged before and after photocrosslinking via scanning electron microscopy (SEM). ChiMA3%GO samples were imaged at 500× and 1500× magnification with a JSM 6490-LV microscope (JEOL, Tokyo, Japan) under vacuum conditions and a 20 kV accelerating voltage. Pore size distribution was determined with the aid of ImageJ^®^ software.

### 2.8. Qualitative Adhesion Test

The adhesion potential of the hydrogels was qualitatively assessed by bonding bone samples in a buffer simulating physiological conditions. Bovine femur fragments were washed by being immersed in PBS 1 × with media changes every 2 h. After 6 h, fragments were dried, cut into cube-shaped specimens, and stored at 4 °C until further use. Then 500 mL of the hydrogels were deposited and photocrosslinked between two bone specimens. The bonded hydrogel–bone complex was then immersed in PBS 1×, incubated at 37 °C, and checked daily until bone samples were fully detached.

### 2.9. Mechanical Adhesion

The mechanical adhesion of the hydrogels was evaluated through the tensile testing of adhesive butt-joint specimens. Bovine femur fragments were washed by immersion in PBS 1X with media changes every 2 h. After 6 h, fragments were dried and cut into dog bone-shaped specimens with a Dremel 4000 moto tool (Bosch Power Tools, Stuttgart, Germany). These specimens were then shopped in half at the center of their longitudinal axis and stored at 4 °C until further use. The viable bonding area of the specimens was determined from microscopy images. Then 500 µL of the hydrogels were deposited and photocrosslinked between the two half-dogbones to assemble the butt-joint specimen. Tensile testing was conducted in a planar uniaxial machine (Bose, Electroforce) with a 500 N load cell at a constant displacement rate of 0.1 mm/s [54].

### 2.10. Texture Analysis

The firmness of the hydrogels was evaluated with a TA.HDplusC texture analyzer (Stable Micro Systems, Godalming, UK) before and after sample irradiation. Hydrogels were molded into 20 mm diameter and 25 mm height cylinder-shaped constructs. This test measured compression force at a 1.0 mm/s speed and 15 mm penetration length using a 10 mm cylindrical probe [49].

### 2.11. Swelling and Degradation

Deposited samples of the hydrogels were weighted, immersed in DMEM supplemented with 10% (*v*/*v*) FBS, and incubated at 37 °C. For swelling, sample weight was monitored every 12 h until no further increase in weight was observed. For degradation, sample weight was monitored every 12 h from maximum weight until the hydrogel was fully dissolved in the medium. The percentage of swelling and degradation was estimated following Equation (2):

(2) $$Swelling\;or\;degradation\;\left(\%\right)=\frac{W_{f}-W_{0}}{W_{0}}\times 100\%\;$$

### 2.12. Hemolysis and Platelet Aggregation

To assess the hemolytic activity of hydrogels, blood samples from a healthy donor were collected in EDTA tubes and centrifuged at 1800 RPM for 5 min to separate the plasma and replace it with PBS. This procedure was repeated until a purified erythrocyte precipitate was obtained. Then 100 mL of a 10% (*v*/*v*) PBS-diluted erythrocyte solution was mixed with 100 mL of each extract and incubated for 1 h at 37 °C. The samples were then centrifuged at 3000 RPM for 5 min, and 100 mL from the supernatant was seeded by triplicate in a 96-well microplate and read at 450 nm in a Multiskan FC microplate reader (Thermo Fisher Scientific, Waltham, MA, USA). Triton 100-X and PBS 1X were used as positive and negative controls, respectively. The percentage of hemolytic activity was estimated following Equation (3):

(3) $$Platelet\;aggregation\left(\%\right)=\frac{Abs_{s}-Abs_{\left(-\right)}}{Abs_{\left(+\right)}-Abs_{\left(-\right)}}\times 100\%$$

For platelet aggregation, blood samples from a healthy donor were collected in sodium citrate tubes and centrifuged at 1000 RPM for 15 min to retrieve the platelet-rich plasma (PRP). Then 100 mL aliquots of each hydrogel were seeded by triplicate with 100 mL of PRP in a 96-well microplate and left under agitation for 1 h. The unaggregated platelets in the supernatant (100 mL) were removed and seeded in a separate microplate with 10 mL of Triton 100-X and left under agitation for 15 min. This microplate was then centrifuged at 1000 RPM for 15 min, and 50 mL of each well was transferred to another microplate. Finally, 50 mL of LDH reagent was pipetted into each of the microplate wells, and the absorbance was then read at 493 nm in a Multiskan FC microplate reader (Thermo Fisher Scientific, Waltham, MA, USA). Platelet aggregation was estimated by comparing absorbances to an LDH calibration curve.

### 2.13. Cytotoxicity

The cytotoxicity of the hydrogels was determined by an indirect contact assay with MTT. First, hydrogel samples were mixed with FBS-free OBM at 25% (*v*/*v*) and incubated for 4 h at 37 °C. Extracts for the assay were obtained by collecting the supernatant of the centrifuged mixtures and preparing dilutions at 25%, 12.5%, 6.25%, 3.13%, and 1.56% (*v*/*v*). Parallelly, 100 µL of NHOst cells was seeded in a 96-well culture plate at a density of 1 × 10^6^ cells/mL and incubated at 37 °C and 5% CO_2_ for 24 h. Culture media were then removed and replaced with FBS-free OBM. Hydrogel extracts were added by triplicate to cell-seeded wells, and the culture plate was then incubated at 37 °C and 5% CO_2_ for another 48 h. Then 10 μL of MTT (5 mg/mL) was added to each well, and cells were incubated at 37 °C and 5% CO_2_ for 2 h. The culture medium was removed, and 100 μL of DMSO was added to dissolve the formed formazan crystals. Absorbance was read at 595 nm in a Multiskan FC microplate reader (Thermo Fisher Scientific, Waltham, MA, USA) [55]. The MTT assay was also performed on Vero cell cultures.

### 2.14. Finite Element Analyses of Bioadhesive-Repaired Bones

Finite element analyses were performed in the software Abaqus/CAE^®^ (Dassault Systemes, Vélizy-Villacoublay, France) to simulate the stress distribution experienced by bioadhesive-repaired femurs during movement. Injured bones were modeled by generating transverse and oblique ruptures on a reconstruction of the distal epiphysis of the femur (obtained from the Embodi 3D repository) in the 3D Slicer^®^ software (The Slicer Community, Boston, MA, USA). To assure mesh convergence, 127,118 and 156,861 C3D4 tetrahedral domain elements were built in the software Ansys^®^ ICEM CFD (Ansys, Canonsburg, PA, USA) for transverse and oblique ruptures, respectively. As an initial resting state, a vertical load of 571 N was distributed over 24 nodes in the medial end of the femur to simulate the patient’s body weight. Additionally, 16 nodes were distributed over the distal end of the femur to represent articular constraints.

**Figure 4:**
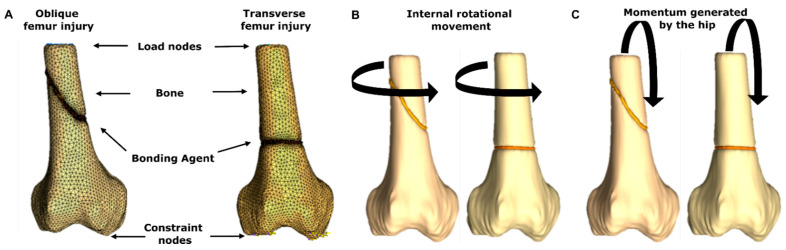
Geometries employed for the FEA simulations. (**A**) Components of the geometries along with the rendered mesh. (**B**) Load direction during internal rotation of the knee. (**C**) Load direction during hip momentum.

The built models were subjected to two kinds of movements: internal rotation of the knee, and momentum from the hip (Figure 4). To simulate knee rotation, a rotational load of 5 N was distributed over 20 nodes in the internal and external flanks of the femur. Similarly, a load of 5 N was exerted diagonally (y+, z−) over the same nodes to simulate hip momentum. The distributions of minimum principal, maximum principal, and von Mises stresses were evaluated for each model. All the simulations were performed with parameters either recovered from the mechanical experiments conducted here or reported for commercial bone cements. Supplementary Table S1 summarizes the mechanical properties of bone tissue, the hydrogel nanocomposite bioadhesive, and bone cement [56,57].

### 2.15. Statistical Analysis

All experimental data were collected in triplicate, for which mean and standard deviation were calculated. Statistical analysis was performed using two-way ANOVA and Tukey’s test for pairwise comparison in GraphPad Prism (GraphPad Software Inc, San Diego, CA, USA). *p* < 0.05 was considered statistically significant.

## 3. Results and Discussion

### 3.1. Chemical Functionalization of Chitosan

The chemical modification of chitosan was confirmed by changes in the FTIR spectrum of ChiMA (Supplementary Figure S1), as the presence of peaks at 1680 cm^−1^ and 670 cm^−1^ corresponded to the C=O stretching and C=C bending vibrations present in the amide-bound methacryloyl groups [58,59]. Peaks at 1580 cm^−1^, 1410 cm^−1^, and 2950 cm^−1^ represented N–H bending, O–H bending, and C–H stretching vibrations, attributed to the glucosamine and N-acetylglucosamine monomers of chitosan [60,61].

XPS survey spectra for the Chitosan sample and ChiMA samples for 1:1, 1:2, and 1:4 molar ratios are shown in Supplementary Figure S2A. The major peaks could be associated with C1s, N1s, and O1s signals, and there was no evidence of chitosan degradation caused either by the XPS setup conditions or the chemical modification process. After this, high-resolution spectra for C, N, and O elements were recorded on the pristine sample and the modified ones under the same setup conditions. The decomposition peaks and corresponding fitting curves for each element and the samples are shown in Supplementary Figure S2B. For the case of C1s, the signal was decomposed into three sub-peak components. The first at lower energy values could be assigned to C–C, C=C, and C–N bonds, the next to C–O– and C–O–C bonds, and the last, at higher energy values to –O–C–O– bonds. For the case of N1s, the signal was also decomposed into three sub-peak components. The first at lower energy values could be assigned to –C–N– bonds, the next to –O–N–C– bonds, and the last to ionic NH+ species, in a good agreement with functional groups existing for chitosan and ChiMA samples. Finally, the O1s signal could be resolved into two sub-peak components attributed to C=O at low binding energy values, and C–O–C or other chemisorbed –C–O– species at higher ones. Results confirmed the successful conjugation of methacryloyl groups in the polymeric backbone of chitosan with the highest yield obtained for the 1:2 ChiMA sample. Moreover, for the elemental analysis quantification, the Relative Sensitivity Factors (RSF) were used to scale the measured peak areas, and the N1s peak area of the 75% deacetylated chitosan sample was selected for a stoichiometric calculation after considering that adventitious carbon is comparative for all samples. The calculated elemental composition of the chitosan and ChiMA samples is summarized in Table 1. Notably, the carbon contents in chitosan seemed to be higher than expected due to the presence of acetyl groups after the required dissolution of chitosan in 1% *v*/*v* acetic acid before conducting the conjugation reaction. The ChiMA 1:1 sample showed a small unidentified sub-peak component at lower binding energy values for C1s and N1s, which can be attributed to surface charge compensation artifacts during the XPS run, which was neglected for the calculations. The increments in the sub-peak components of carbonyl and amide groups relative to unmodified chitosan are summarized in Table 2. These findings are consistent with previous reports in the literature and support the notion that the 1:2 ratio provides the highest methacrylation level [41].

### 3.2. Synthesis of Graphene Oxide

**Figure 5:**
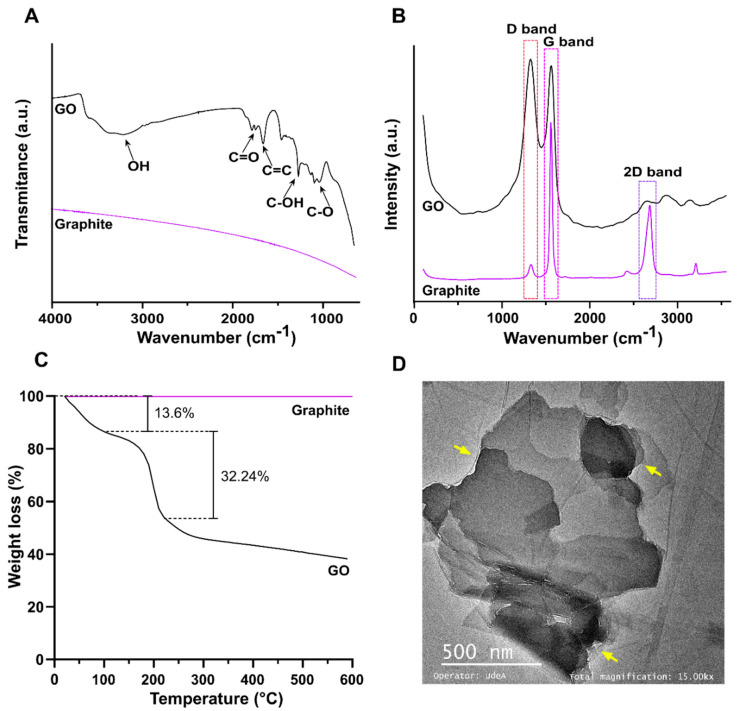
Physicochemical characterizations of GO. (**A**) FTIR spectra of chitosan, graphite, and GO. (**B**) Raman spectra of graphite and GO. (**C**) TGA of GO. (**D**) TEM of GO.

The correct synthesis and oxidation level of GO were confirmed by FTIR, Raman spectroscopy, TGA, and TEM analyses, as shown in Figure 5. The FTIR spectra (Figure 5A) proved the correct oxidation of graphite, as GO exhibits multiple peaks related to oxidative functional groups [62,63]. Peaks at 3392 cm^−1^ and 1226 cm^−1^ corresponded to O–H and C–OH stretching, while peaks at 1740 cm^−1^ and 1050 cm^−1^ represented C=O stretching and C–O bending, respectively. The peak at 1620 cm^−1^ could be attributed to C=C aromatic stretching. The absence of peaks near 1250 cm^−1^ confirmed that the material was not partially reduced into reduced graphene oxide (rGO) [50,62,63,64].

The Raman spectrum (Figure 5B) of graphite presented strong G and 2D bands at 1570.8 cm^−1^ and 2705.1 cm^−1^, while the spectrum of GO showed strong G and D bands at 1588.9 cm^−1^ and 1345.8 cm^−1^ [65,66]. The G band, which relates to the sp2 hybridization in carbon, most likely shifted to 1588.9 due to the oxidation of graphite [65]. Similarly, the noticeable growth of the D band, which results from vacancies or dislocations that disrupt sp2 carbon layers, suggests the transition of some carbon atoms in graphene to and sp3 hybridization to accept functional groups [67]. The I(D)/I(G) ratio for GO corresponded to 1.03, which was most likely due to a high oxidation level [64]. Furthermore, the weakening of the 2D band can be explained by breaking the stacking order along the c-axis in graphite due to the oxidation reaction, thus confirming the high level of oxidation in the synthesized GO [64]. Similarly, the I(2D)/I(G) ratio for GO corresponded to 0.4, demonstrating the graphene-like structure of the synthesized material [68].

The TGA thermogram of GO (Figure 5C) presented three noticeable weight losses. A first weight loss of 13.6% from room temperature to 100 °C could be attributed to the evaporation of bound water. A second weight loss of 32.24% between 100 and 200° could be attributed to removal of oxygen-rich species such as C–O, C–OH, and C=O, further confirming the high level of oxidation of the synthesized GO [69,70]. The TEM micrograph of GO nanosheets (Figure 5D) demonstrated the characteristic flake-like structure of the material [71]. Different levels of opacity in the image revealed non-uniform stacking of multiple GO layers. Regions of higher transparency indicated thinner films, while darker regions indicated greater stacking [72]. Moreover, the disordered and unwrinkled structure of the GO sheets could be attributed to the high abundance of oxygen-rich functional groups on the surface [73].

### 3.3. Rheological Evaluation

**Figure 6:**
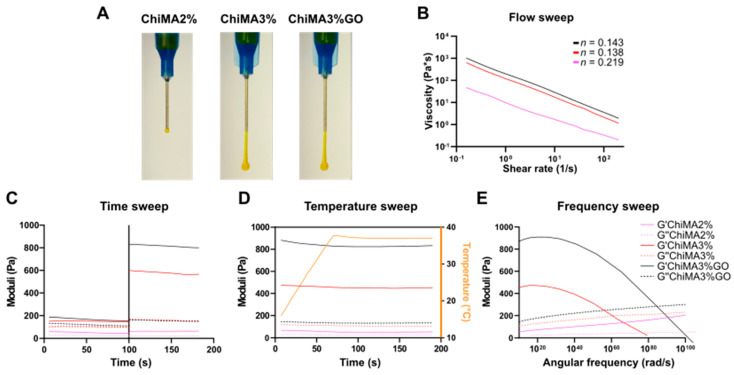
(**A**) Manual injection of the hydrogels through a 21-gauge needle. (**B**) Flow sweep of photocrosslinked samples. (**C**) Time sweeps before and after blue-light photocrosslinking. (**D**) Temperature sweep of photocrosslinked samples between 5 °C and 37 °C. (**E**) Frequency sweep of photocrosslinked samples.

Three potential formulations for the bioadhesive were successfully prepared: ChiMA2%, ChiMA3%, and ChiMA3%GO. These hydrogels presented an apparent homogeneous consistency and integrity, suggesting the materials’ potential to endure mild alkaline pH adjustments instead of regular chitosan, which deprotonates and aggregates into insoluble clusters [51]. Consequently, all formulations allowed extrusion through a 21-gauge blunt needle, although only ChiMA3% and ChiMA3%GO could form continuous filaments, while ChiMA2% dripped ununiformly, forming drops due to low viscosity and, consequently, lack of consistency (Figure 6A).

Figure 6B–E present the changes in the storage modulus (G′) and loss modulus (G′′) of the hydrogels when subjected to different stimuli. The flow, temperature, and frequency experiments were performed on irradiated samples, as exposure to blue light (Figure 6C) significantly improved both moduli, favoring mechanical stability and a solid-like behavior [51,60]. The flow sweep (Figure 6B) shows that the hydrogels exhibited lower viscosity at higher shear rates, characteristic of pseudoplastic fluids. This shear-thinning behavior could be further confirmed by the power-law fittings, shown in Supplementary Figure S3, yielding values of *n* < 1 for all formulations [60,74]. Moreover, temperature increments between 15 °C and 37 °C (Figure 6D) caused a slight decrease in both G′ and G′′, implying that less energy was required for deformation and thus suggesting the weakening of crosslinking bonds [75,76]. However, according to an ANOVA one-way test, these temperature-induced changes in the moduli were non-statistically significant (*p*-value > 0.9999). Furthermore, the frequency sweep test (Figure 6E) showed that both moduli were frequency-dependent, as evidenced by the decrease of G′ and the increase of G′′ with increasing angular frequency. Hydrogels maintained a predominant solid-like behavior until G′ crosses G′′ (gel point), after which they acquired a predominant liquid-like behavior [77].

In general, all hydrogels presented a higher G′ and a lower G′′, confirming the material’s potential to store deformation energy with small amounts of dissipation from internal friction [60]. The ChiMA2% formulation yielded low G′ and G′′, suggesting that ChiMA’s concentration was insufficient to form a mechanically stable polymer network. In contrast, ChiMA3% presented desirable values for both moduli, exhibiting a sustained response to the studied stimuli. ChiMA3%GO yielded the highest G′, confirming that the addition of GO to the hydrogels significantly improves their mechanical strength, however potentially compromising their swelling capacity [78].

### 3.4. GO Dispersion and Morphological Structure of the Hydrogels

**Figure 7:**
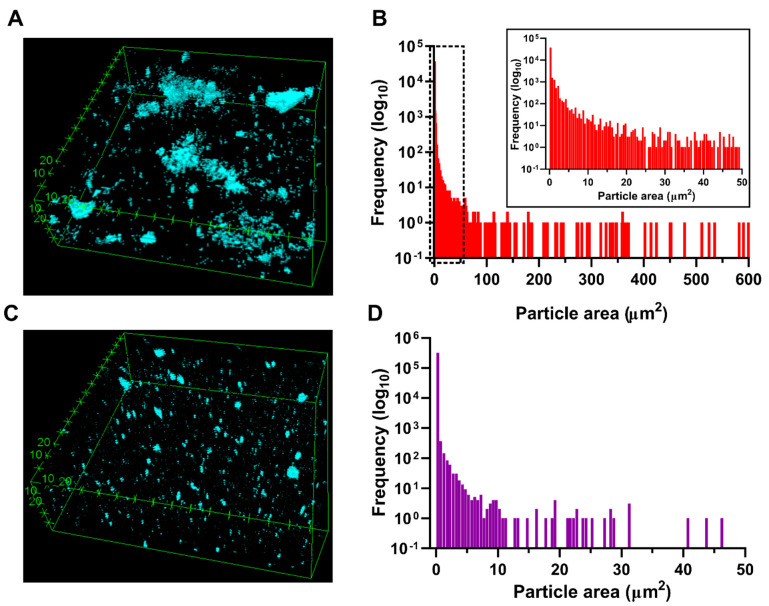
Dispersion of GO and morphological structure of the nanocomposite hydrogel. (**A**) Dispersion of serum-free GO in a ChiMA matrix. (**B**) Distribution of serum-free GO particle area, which shows proper tail distribution centered at 0.386 mm^2^. (**C**) Dispersion of serum-doped GO in a ChiMA matrix. (**D**) Distribution of serum-doped GO particle area, which shows proper tail distribution centered at 0.024 mm^2^.

Dispersion of GO in the ChiMA hydrogel matrix was observed in Z-stack reconstructions from confocal microscopy images. As evidenced in Figure 7A,B, the nanomaterial’s dispersion was limited by its own aggregation, as the particle area distribution exhibited a right tail centered at 0.386 µm^2^, but with agglomerates as large as 566.805 µm^2^. To address this issue, we sought to promote the adsorption of hydrophilic serum proteins from culture media to the surface of GO nanosheets, as proposed by Rueda-Gensini et al. [53]. Thus, GO was mixed with FBS-supplemented DMEM before its addition to the ChiMA pre-gel, yielding the homogeneous dispersion presented in Figure 7C,D. In this case, the particle area distribution exhibited a right tail centered at 0.024 µm^2^ with agglomerates as large as 44.640 µm^2^, which corroborates a much higher dispersion of GO in the ChiMA hydrogels [53].

Similarly, the morphology of the polymeric matrix in the ChiMA3%GO hydrogel was observed before and after photocrosslinking via SEM, as shown in Supplementary Figure S4. Under the effect of blue light, riboflavin degrades and generates free radicals that destabilize the alkene bonds in ChiMA, thus inducing covalent crosslinking between adjacent strands and causing the microstructure to collapse into smaller pores [74]. Differences in size were visually perceptible and statistically significant, as pore diameter varied between 2.9 µm and 28.2 µm, with a mean of 13.4 µm for non-irradiated samples. In comparison, irradiated samples varied from 0.4 µm to 12.7 µm with an average of 3.3 µm. However, crosslinked samples also exhibited an uneven microstructure, which could be attributed to the formation of imine bonds between GO and the free amine radicals in unmethacrylated units of N-acetyl glucosamine [51,79,80].

### 3.5. Mechanical and Adhesion Evaluation

**Figure 8:**
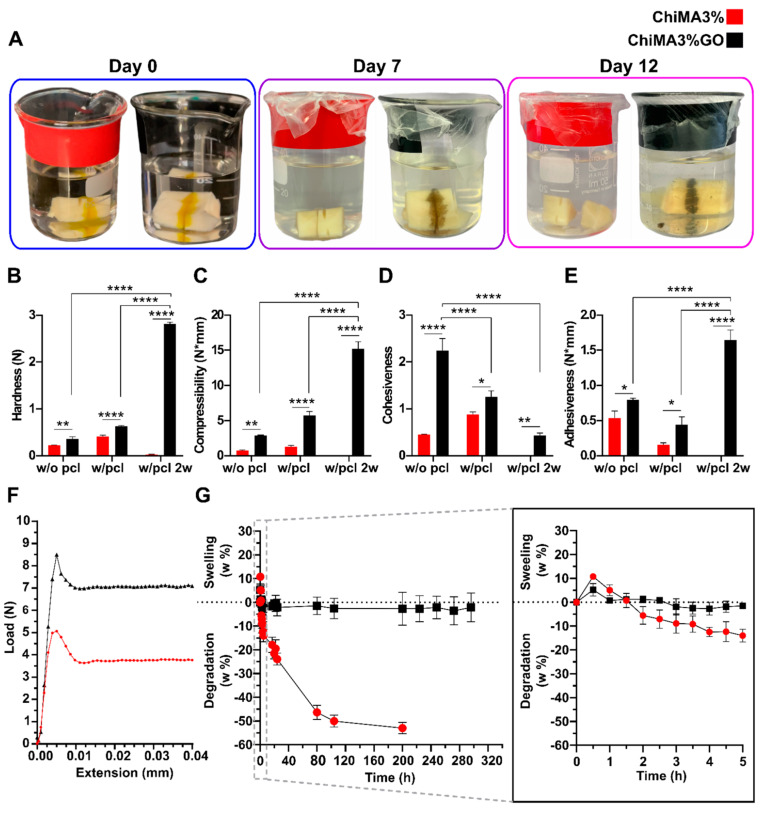
Mechanical and adhesive evaluation of the ChiMA3% and ChiMA3%GO hydrogels. (**A**) Qualitative adhesion of bone specimens at 0, 7, and 12 days. (**B**) Hardness, (**C**) compressibility, (**D**) cohesiveness, and (**E**) adhesiveness of the hydrogels before and after photocrosslinking and 2-weeks of incubation at 37 °C (two-way ANOVA * *p* ≤ 0.05 ** *p* ≤ 0.01 *** *p* ≤ 0.001 **** *p* ≤ 0.0001). (**F**) Tensile strength of butt-joint specimens. (**G**) Swelling and degradation of the hydrogels.

The results for the qualitative and quantitative evaluation of the hydrogels’ adhesion are presented in Figure 8. Initially, adhesion was qualitatively assessed by bonding two bone fragments in a buffer simulating physiological conditions. The ChiMA3% hydrogel maintained adhesion under wet conditions for only ten days, while the ChiMA3%GO hydrogel successfully maintained bonding for over two weeks. Moreover, this hydrogel progressively darkened for days, thus reflecting GO’s reduction in the warm environment (Figure 8A). Further texture analysis was performed with the hydrogels encompassing hardness, compressibility, cohesiveness, and adhesiveness measurements. Hardness, determined as the maximum peak force during the first compression cycle, was studied to estimate the required force to produce deformation of the gels [81]. As evidenced in Figure 8B, ChiMA3%GO samples yielded significantly higher values (*p* < 0.01) than the ChiMA3% samples, confirming GO’s contribution to the structural stability of the hydrogels. Moreover, hardness was significantly improved by the crosslinking stimuli, mainly by GO’s reduction during incubation. This shift from low to high hardness is a desirable behavior, as it indicates that unstimulated samples will be easily spreadable, while after stimulation, the hydrogel will be retained on the surface [82]. The hardness of ChiMA3% was null after two-weeks of incubation because of the sample degradation. Compressibility (Figure 8C) was estimated as the work required to deform the material during the first compression [81]. This property was significantly enhanced by irradiation and incubation for the ChiMA3%GO samples, favoring the material’s potential to endure greater compression loads. Once again, the shift from low to high values increased spreadability and resistance before and after irradiation, respectively [81]. The differences between the GO-free samples were non-statistically significant.

Cohesiveness reflects the reconstruction ability of gels after application and was determined as the ratio of the area under the force–time curve on the second compression cycle to that produced on the first one (Figure 8D) [81]. The addition of GO into the hydrogel significantly improved the material’s cohesiveness, thus favoring its manipulation. However, cohesiveness was reduced during both crosslinking stages, limiting the structural recovery of the samples [83].

Adhesiveness represents the work required to overcome the attractive forces between the surfaces of the hydrogel and the probe and was estimated as the negative force area for the first compression cycle (Figure 8E) [81]. The addition of GO significantly increases the hydrogel’s stickiness, thus improving its chances of remaining attached to the surface of bones [84]. Although this property was partially reduced after crosslinking, it was largely recovered after incubation. This final improvement of adhesiveness suggests that the hydrogel’s application could potentially shorten the treatment length and improve patient compliance [85].

Finally, a mechanical tensile test was made to evaluate the adhesive’s performance over butt-joined specimens. As evidenced in Figure 8F, ChiMA3% can withstand 5 N loads. At the same time, ChiMA3%GO can endure over 8.5 N. These values are well within the range reported for other bioadhesives and further confirm that the addition of GO significantly improves ChiMA’s capacity to bear tensile loads.

### 3.6. Swelling and Degradation

Figure 8G shows the swelling and degradation profiles of both hydrogels under the simulated physiological conditions. Although limited, both hydrogels achieved their maximum swelling capacity within the first hour, yielding maximum differences of 10.73% for ChiMA3% and 5.06% for ChiMA3%GO. Similarly, results showed that over 50% of the ChiMA3% hydrogel was degraded within two weeks, while the ChiMA3%GO hydrogel seems unaffected by exposure to the medium. These results support the notion that the addition of GO into the ChiMA matrix enhances its mechanical stability, although at the expense of its swelling capacity. However, although this limited swelling restricts the potential of loading functional molecules within the microstructure’s pores, immobilization or adsorption on the GO’s surface could be alternatively exploited for controlled drug delivery in future applications.

### 3.7. Hemolysis and Platelet Aggregation

**Figure 9:**
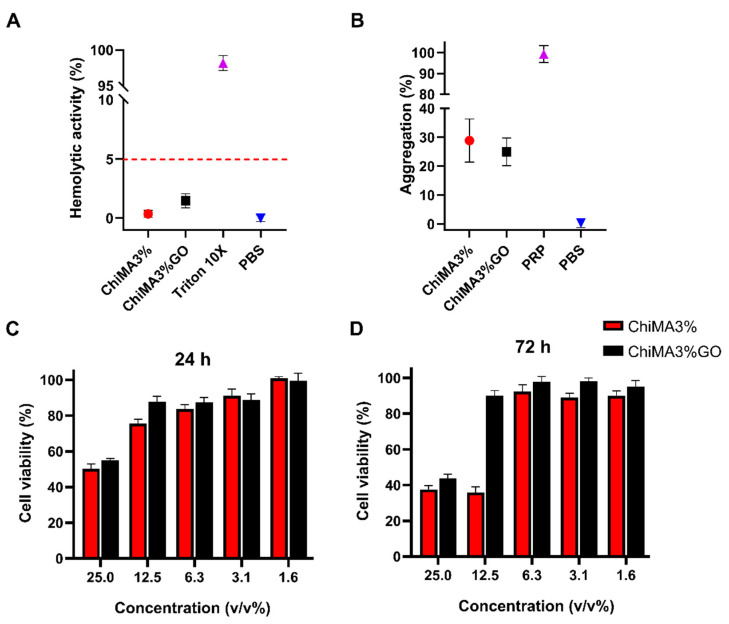
Biocompatibility evaluation of the ChiMA3% and ChiMA3%GO hydrogels. (**A**) Hemolytic activity and (**B**) platelet aggregation of the adhesive hydrogels. Cell viability of NHOst cells when exposed to the hydrogels for (**C**) 24 h and (**D**) 72 h.

Both hydrogels presented a non-hemolytic behavior, as the hemolysis percentage approached 1% for ChiMA3% and 2% for ChiMA3%GO (Figure 9A). Despite GO’s reported dose-dependent hemolytic activity, its incorporation into a biocompatible polymer matrix such as chitosan might have helped reduce such a tendency [86,87]. Similarly, platelet aggregation was estimated to be 28.89% for ChiMA3% and 20.48% for ChiMA3%GO, implying that they can both be classified as intermediate aggregates (Figure 9B). Aggregation is favorable for bone regeneration, as platelets promote migration and proliferation of osteogenic cells, increase blood vessel formation, and induce inflammatory reactions [88,89]. Although higher aggregation was expected, recent reports have suggested that methacryloyl modifications may inhibit platelet aggregation by overshadowing the polymeric backbone of chitosan [90,91].

### 3.8. Cytotoxicity

Results from the MTT assays revealed that the hydrogels exhibited promising cytocompatibility (Figure 9C,D). None of the evaluated concentrations yielded cell viability levels below 50% after 24 h and 30% after 72 h for the NHOst cells. Moreover, viability increased as sample concentration decreased, reaching over 80% for 6.3% (*v*/*v*) concentrations and below. This sharp shift in viability suggests that the material induces cytotoxicity in a dose-dependent manner. In addition, microplate in vitro assays may broadly predict material–cell culture interactions but fail to represent clearance phenomena, and thus some hydrogel components such as riboflavin may accumulate in a non-representative population of cells [92]. Importantly, viability appeared to be enhanced in samples with GO, suggesting that its addition may impede the diffusion and accumulation of riboflavin in the culture medium. This can happen both by ChiMA3%GO’s smaller porosity and ChiMA3%’s unrestricted degradation. Similar results were obtained for the Vero cell cultures (Supplementary Figure S6).

### 3.9. Simulations

**Figure 10:**
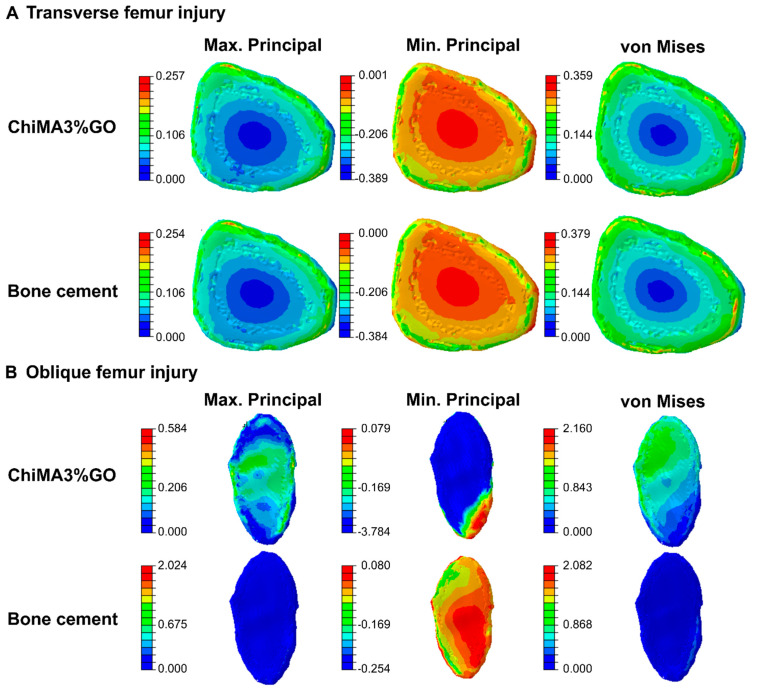
Distribution of tensile and compressive forces over ChiMA3%GO- and bone cement-repaired femurs under the effect of internal knee rotation. Maximum principal, minimum principal, and von Mises stresses are shown for (**A**) the transverse lesion and (**B**) the oblique lesion.

**Figure 11:**
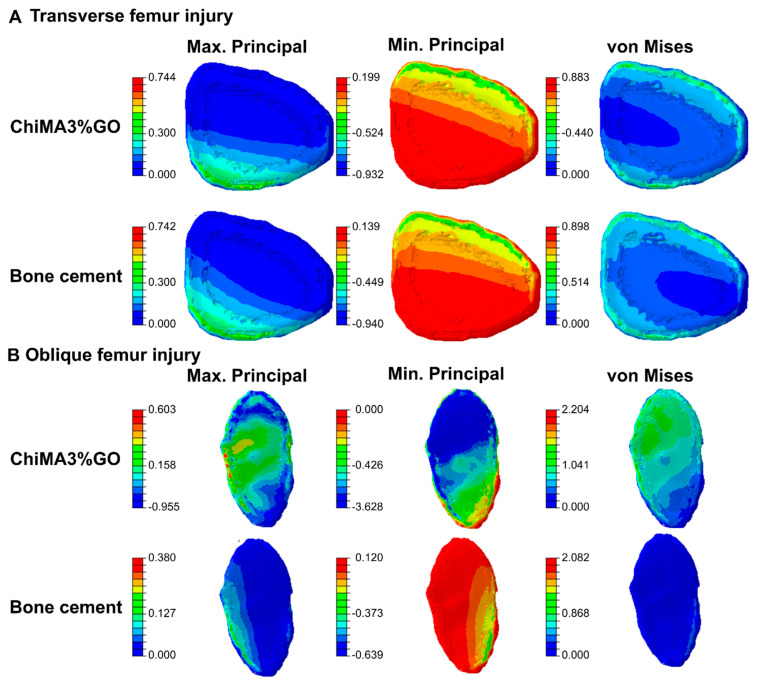
Distribution of tensile and compressive forces over ChiMA3%GO- and bone cement-repaired femurs under the effect of hip momentum. Maximum principal, minimum principal, and von Mises stresses are shown for (**A**) the transverse lesion and (**B**) the oblique lesion.

Simulation results for the knee rotation and hip momentum models are shown in Figure 10 and Figure 11. The transverse lesion (Figure 10A) showed increased values for the minimum principal (9.88%) and maximum principal (1.18%) stresses and a decrease in the von Mises stress (−5.28%). In contrast, the oblique lesion in knee rotation (Figure 10B) yielded increased values for the minimum principal (1389.76%), maximum principal (17.98%), and von Mises (554.55%) stresses with respect to the commercial bone cement. Additionally, the transverse lesion in hip momentum (Figure 11A) yielded a decrease in the minimum principal (−5.67%), maximum principal (−5.34%), and the von Mises (−0.79%) stresses. On the contrary, the oblique lesion (Figure 11B) exhibited increased values for the minimum principal (467.76%), maximum principal (58.68%), and von Mises (279.35%) stresses.

The obtained results revealed that the femur distribution of tension and compression forces was similar for both treatments. However, during internal rotation, the presence of the bioadhesive led to a more uniform distribution when compared with the bone cement, thereby avoiding the generation of stress concentrators. The absence of such areas prevents ruptures in the bonding material, which favors the adhesion’s endurance. Moreover, by generating a more uniform distribution of loads, the bioadhesive can enhance the healing process by enabling the continuous transmission of forces and mechanical signals between the cells present in the bonded bone segments [93]. Young’s moduli differences explain changes in the distribution of tension and compression forces between the two treatments. The bioadhesive’s relative improved elasticity can be beneficial for bone repair, as it prevents stress shielding. This phenomenon arises from tissue adaptation to reduced loads and leads to bone density loss [94]. By putting these results together, it is possible to confirm the bioadhesive’s suitability for bone tissue repair applications.

## 4. Conclusions

Here, we put forward a ChiMA/GO photoresponsive and adhesive nanocomposite hydrogel to overcome major issues of conventional bone adhesives, i.e., low biocompatibility, poor performance under physiological conditions, difficult applicability, and limited possibilities for tuning mechanical properties in situ. Addition of GO to ChiMA hydrogels led to improved mechanical integrity and resistance, which were crucial for withstanding bone-to-bone adhesion in a simulated physiological medium for over two weeks. XPS and FTIR analyses along with SEM images allowed us to correlate photo-crosslinking by short blue light exposure at room temperature, with key performance attributes such as pseudoplastic response, superior textural properties, and tensile resistance of about 8.5 N. Cytocompatibility assays corroborated cell viability levels above 80% for concentrations below 6% (*v*/*v*) of the hydrogel. The material showed intermediate platelet aggregation, which is advantageous for enhanced tissue regeneration. In addition, the hydrogels exhibited hemolytic tendencies well below 5%. In addition to these major attractive attributes, we demonstrated that the materials could be spread easily on surfaces and tended to remain attached to them. Finally, in silico studies showed the superior performance of the bioadhesives in femur fractures when compared to a commercially available cement for both oblique and transverse lesions. This was attributed to the hydrogel’s ability to distribute the applied loading more uniformly. Even though the developed formulations incorporated relatively well exfoliated GO, future work should focus on maximizing the dispersion, as it has been demonstrated to be a key parameter to assure a much higher material stability. This is a critical attribute, as it directly impacts load distribution and resistance to wet conditions, and the possibility for more uniform bone repair processes enabled by osteoinduction/osteoconduction induced by GO distributed homogeneously at the bone-to-bone interfaces.

We are confident that the results put forward here pave the way for developing new functional nanocomposite hydrogels for applications in orthopedics. Next steps will be dedicated to exploring the treatment of bone lesions in vivo. This will be a major milestone to assure moving from the bench to the bedside in the near future.

## Figures and Tables

**Table 1:** Surface chemical analysis and elemental composition (%) of the chitosan and ChiMA 1:1, 1:2, and 1:4 samples. Details of C1s, N1s, and O1s decomposed peaks and related binding energies (BE) and FWHM values.

Sample	Peak	BE (eV)	FWHM	Corrected Area RSF	Ratio (%)
ChiMA	N1s	398.83 399.55 400.98	1.08 1.04 1.84	842	6.10
	C1s	284.57 286.07 287.78	1.16 1.43 1.31	8594	62.28
	O1s	531.58 532.44	1.36 1.4	4835	31.62
ChiMA 1:1	N1s	398.78 399.42 401.09	1.25 1.34 2.19	698	6.61
	C1s	284.56 285.97 287.57	1.28 1.41 1.29	5884	55.71
	O1s	530.93 532.16	1.89 1.51	3980	37.68
ChiMA 1:2	N1s	398.91 399.53 401.45	1.46 1.63 1.43	728	7.59
	C1s	284.67 286.21 287.82	1.42 1.42 1.49	5390	56.17
	O1s	531.62 532.53	1.85 1.41	3478	36.24
ChiMA 1:4	N1s	398.69 399.41 401.22	1.43 1.36 1.69	864	7.36
	C1s	284.54 286.10 287.68	1.43 1.41 1.33	6827	58.16
	O1s	531.35 532.39	1.78 1.41	4048	34.48

ChiMA: chitosan methacrylate.

**Table 2:** Semi-quantification of sub-peak component variation (%) for carbonyl and amide groups in ChiMA samples with respect to the pristine chitosan.

	ChiMA 1:1	ChiMA 1:2	ChiMA 1:4
Carbonyl sub-peak (D%)	24	30	16
Amide sub-peak (D%)	17	21	12

ChiMA: chitosan methacrylate.

## Supplementary Materials

The following are available online at https://www.mdpi.com/article/10.3390/polym14010126/s1, Table S1. Material properties of bone, bioadhesive, and bone cement, Figure S1: FTIR of graphite, graphene oxide, chitosan methacrylate (ChiMA), and chitosan methacrylate mixed with graphene oxide (ChiMAGO), Figure S2: XPS spectra of ChiMA samples to assess the degree of methacrylation, Figure S3: Flow sweep power-law model fitting, Figure S4: SEM imaging of the hydrogel structure. Figure S5: Platelet aggregation calibration curve, Figure S6: Cell viability of Vero cells when exposed to the hydrogels for (A) 24 h and (B) 72 h, Figure S7: Distribution of tensile and compressive forces over ChiMA3%-, ChiMA3%GO-, and bone cement-repaired femurs under the effect of internal knee rotation, Figure S8: Distribution of tensile and compressive forces over ChiMA3%, ChiMA3%GO, and bone cement-repaired femurs under the effect of hip momentum.
