# Supplementary material for: Preparation and Characterization of an Injectable and Photo-Responsive Chitosan Methacrylate/Graphene Oxide Hydrogel: Potential Applications in Bone Tissue Adhesion and Repair
Source: Polymers (Basel). 2021 Dec 30;14(1):126. doi: 10.3390/polym14010126 (PMC8747203; doi:10.3390/polym14010126)
Supplement: Supplementary file 1 [file polymers-14-00126-s001.zip › polymers-1509769-supplementary.pdf]

Supplementary material

Preparation and characterization of an injectable and photo-responsive chitosan methacrylate/graphene oxide hydrogel: potential applications in bone tissue adhesion and repair

Daniela N. Céspedes-Valenzuela [1,†], Santiago Sánchez-Rentería [1,†], Javier Cifuentes [1], Mónica Gantiva-Diaz [1,2], Julian A. Serna [1], Luis H. Reyes [3], Carlos Ostos [4], Christian Cifuentes-De la Portilla [2], Carolina Muñoz-Camargo [1,*], and Juan C. Cruz [1,*]

[1] Grupo de Investigación en Nanobiomateriales, Ingeniería celular y Bioimpresión (GINIB), Department of Biomedical Engineering, Universidad de los Andes, Bogotá 111711, Colombia; dn.cespedes@uniandes.edu.co (D.N.C.); s.sanchezr2@uniandes.edu.co (S.S.); jf.cifuentes10@uniandes.edu.co (J.C.); mr.gantiva@uniandes.edu.co (M.G.); ja.serna10@uniandes.edu.co (J.A.S.)
[2] Grupo de Investigación en Biomecánica (IBIOMECH), Department of Biomedical Engineering, Universidad de los Andes, Bogotá 111711, Colombia; mr.gantiva@uniandes.edu.co (M.G.); cc.cifuentes@uniandes.edu.co (C.C.)
[3] Department of Chemical and Food Engineering, School of Engineering, Universidad de Los Andes, Carrera 1 No. 18A-12, 111711 Bogotá, Colombia; lh.reyes@uniandes.edu.co (L.H.R.)
[4] Grupo CATALAD, Instituto de Química, Universidad de Antioquia, Medellín 050010, Colombia; carlos.ostos@udea.edu.co (C.O.)
* Correspondence: c.munoz2016@uniandes.edu.co (C.M.-C.); jc.cruz@uniandes.edu.co (J.C.C.); Tel.: +57-1-339-4949 (ext. 1789) (J.C.C.)
† These authors contributed equally to this work.

Citation: Céspedes-Valenzuela, D.N.; Sánchez-Rentería, S.; Cifuentes, J.; Gantiva-Diaz, M.; Serna, J.A.; Reyes, L.H.; Ostos, C.; Portilla, C.C.-D.l.; Muñoz-Camargo, C.; Cruz, J.C. Preparation and Characterization of an Injectable and Photo-Responsive Chitosan Methacrylate/Graphene Oxide Hydrogel: Potential Applications in Bone Tissue Adhesion and Repair. *Polymers* **2022**, *14*, 126. https://doi.org/10.3390/polym14010126

Academic Editors: Antonio M. Borrero-López, Concepción Valencia-Barragán, Esperanza Cortés Triviño, Adrián Tenorio-Alfonso and Clara Delgado-Sánchez

Received: 30 November 2021
Accepted: 16 December 2021
Published: 30 December 2021

Publisher's Note: MDPI stays neutral with regard to jurisdictional claims in published maps and institutional affiliations.

Copyright: © 2021 by the authors. Submitted for possible open access publication under the terms and conditions of the Creative Commons Attribution (CC BY) license (https://creativecommons.org/licenses/by/4.0/).

Table S1. Material properties for bone, the hydrogel bioadhesive and bone cement.

Material	Young's modulus (MPa)	Poisson's ratio
Bone	17000	0.3
ChiMA3%	1700	0.3
ChiMA3%GO	2300	0.3
Bone cement	3300	0.3

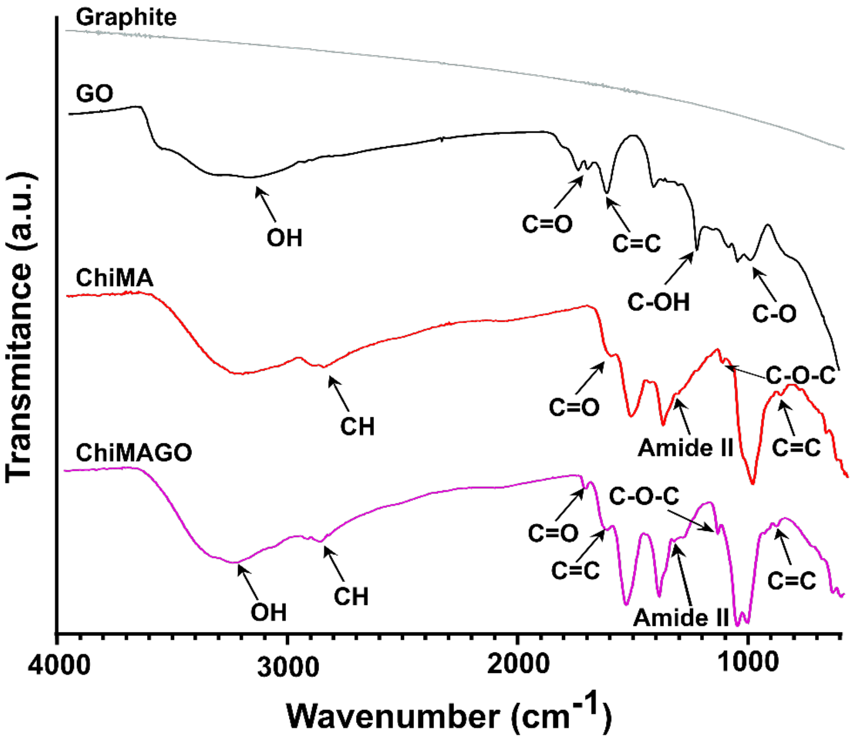

Figure S1. FTIR of graphite, graphene oxide, chitosan methacrylate (ChiMA) and chitosan methacrylate mixed with graphene oxide (ChiMAGO).

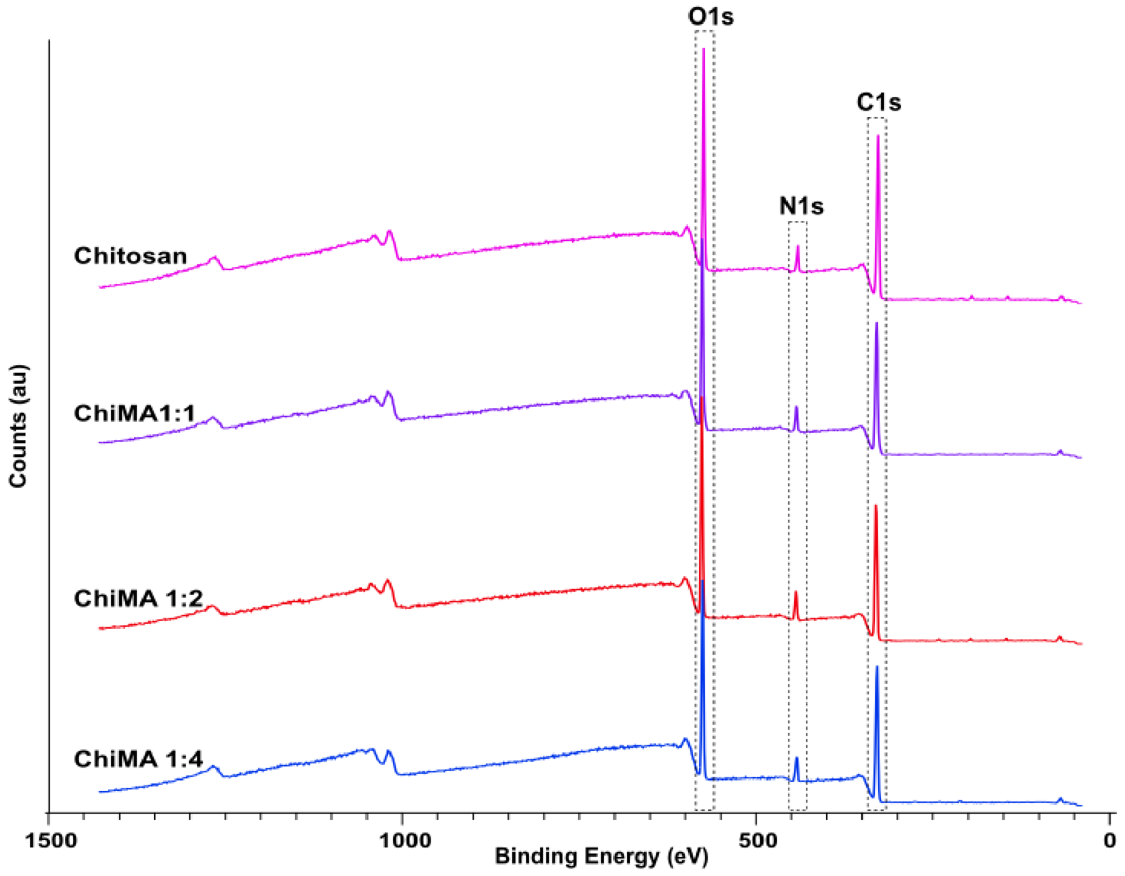

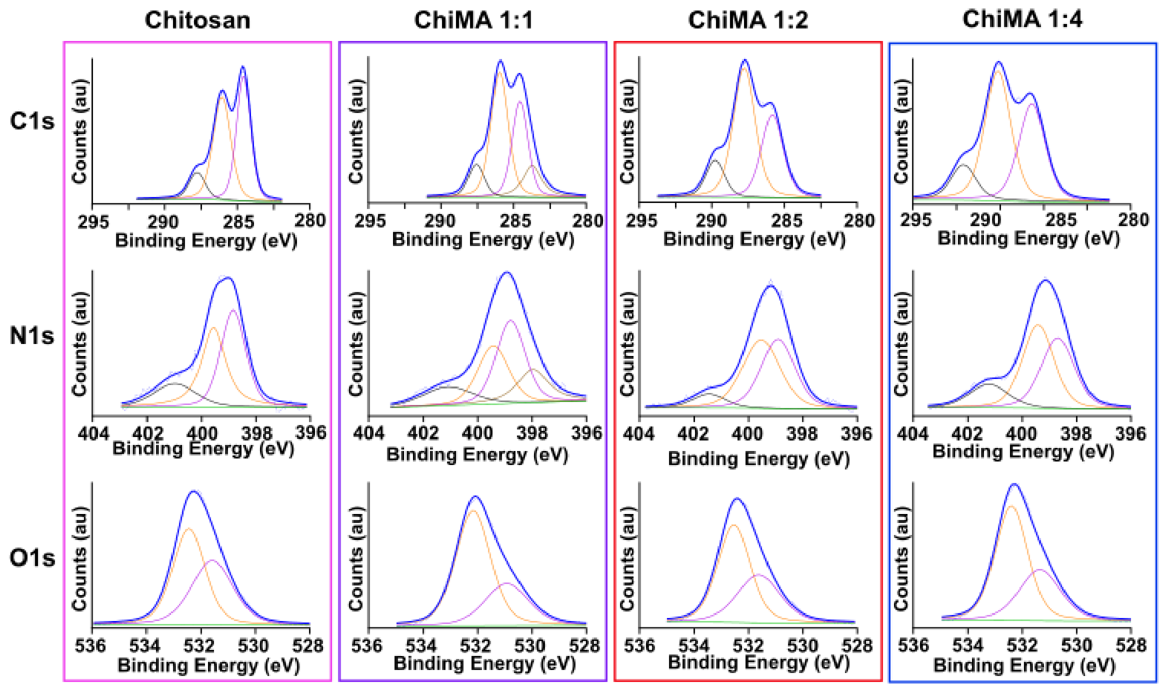

Figure S2. XPS spectra of ChiMA samples to assess the degree of methacrylation. (A) XPS narrow scan spectra of chitosan and ChiMA 1:1, 1:2 and 1:4 samples for C1s, N1s and O1s signals. (B) The sub-peak components are shown under the fitted curves (blue line).

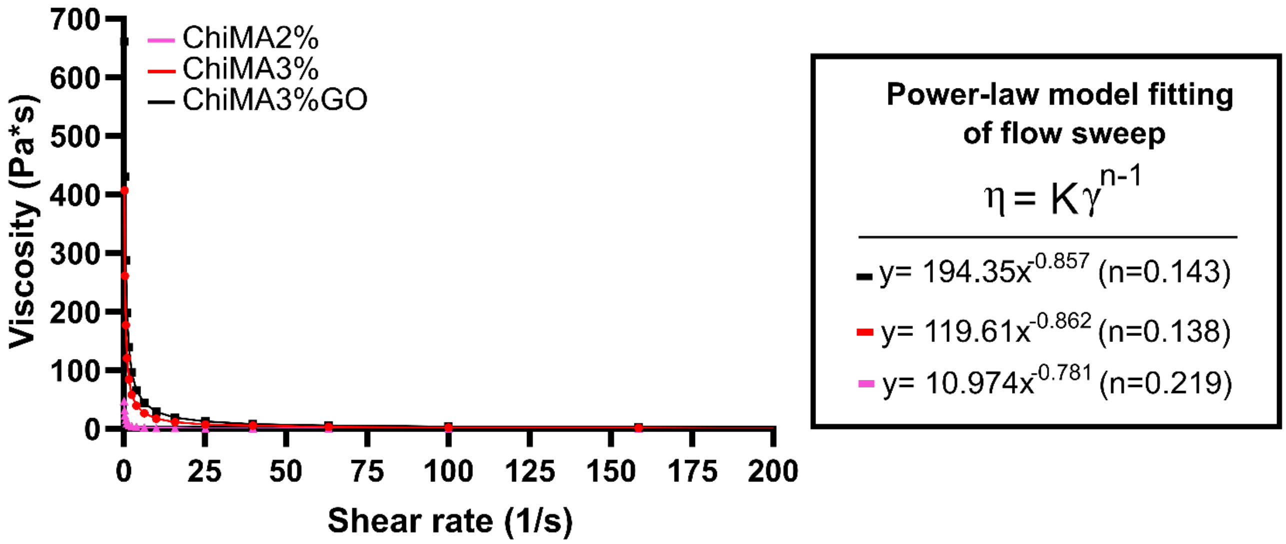

Figure S3. Flow sweep power-law model fitting. Flow sweep profiles of ChiMA2%, ChiMA3% and ChiMA3%GO were fitted to a power-law model to find the corresponding n coefficient, which is indicative of their shear thinning behavior. n values close to 0.2 indicate a strong shear-thinning response.

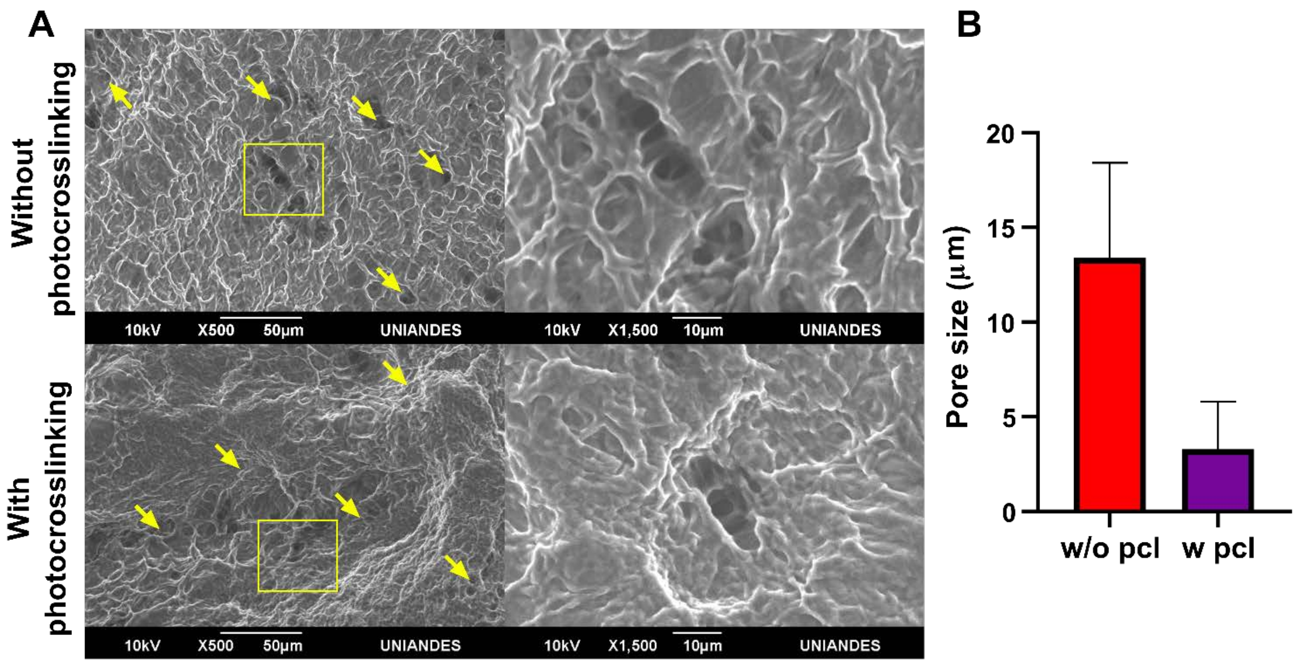

Figure S4. SEM imaging of the hydrogel structure. (**A**) SEM images of the microporous structure of the ChiMA3%GO matrix with and without photocrosslinking. **(B)** Average pore diameter in the ChiMA3%GO matrix with and without photocrosslinking.

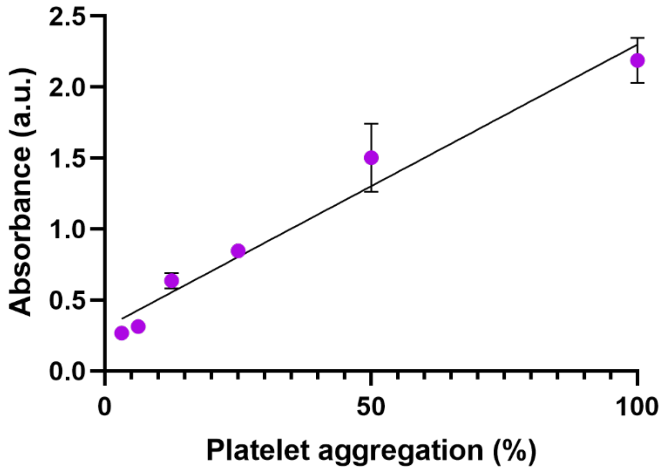

Figure S5. Platelet aggregation calibration curve.

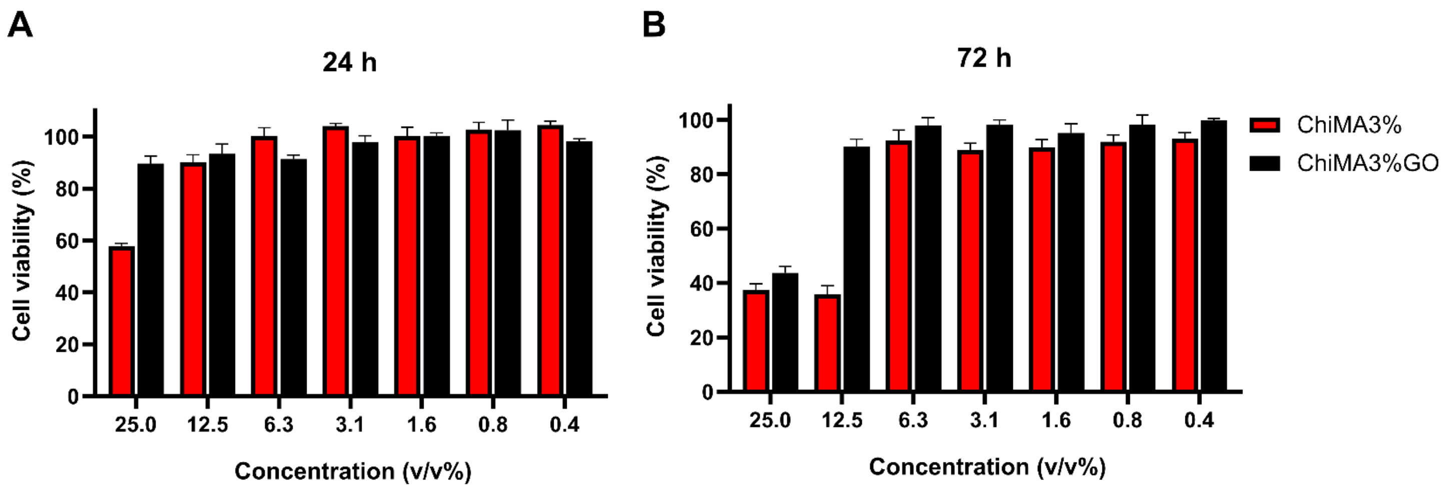

Figure S6. Cell viability of Vero cells when exposed to the hydrogels for **(A)** 24 hours and **(B)** 72 hours.

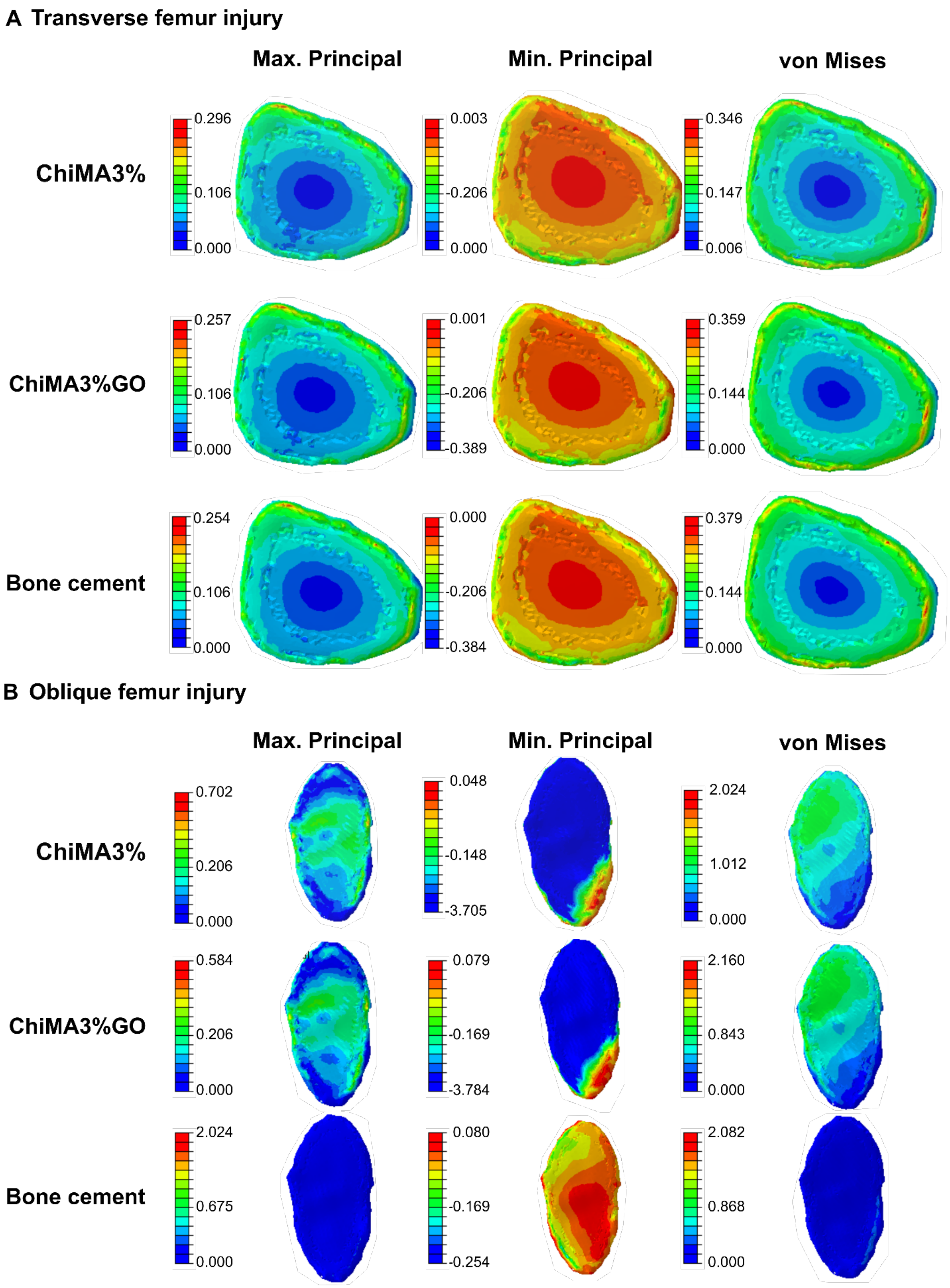

Figure S7. Distribution of tensile and compressive forces over ChiMA3%-, ChiMA3%GO- and bone cement-repaired femurs under the effect of internal knee rotation. Maximum principal, minimum principal, and von Mises stresses are shown for **(A)** the transverse lesion and **(B)** the oblique lesion

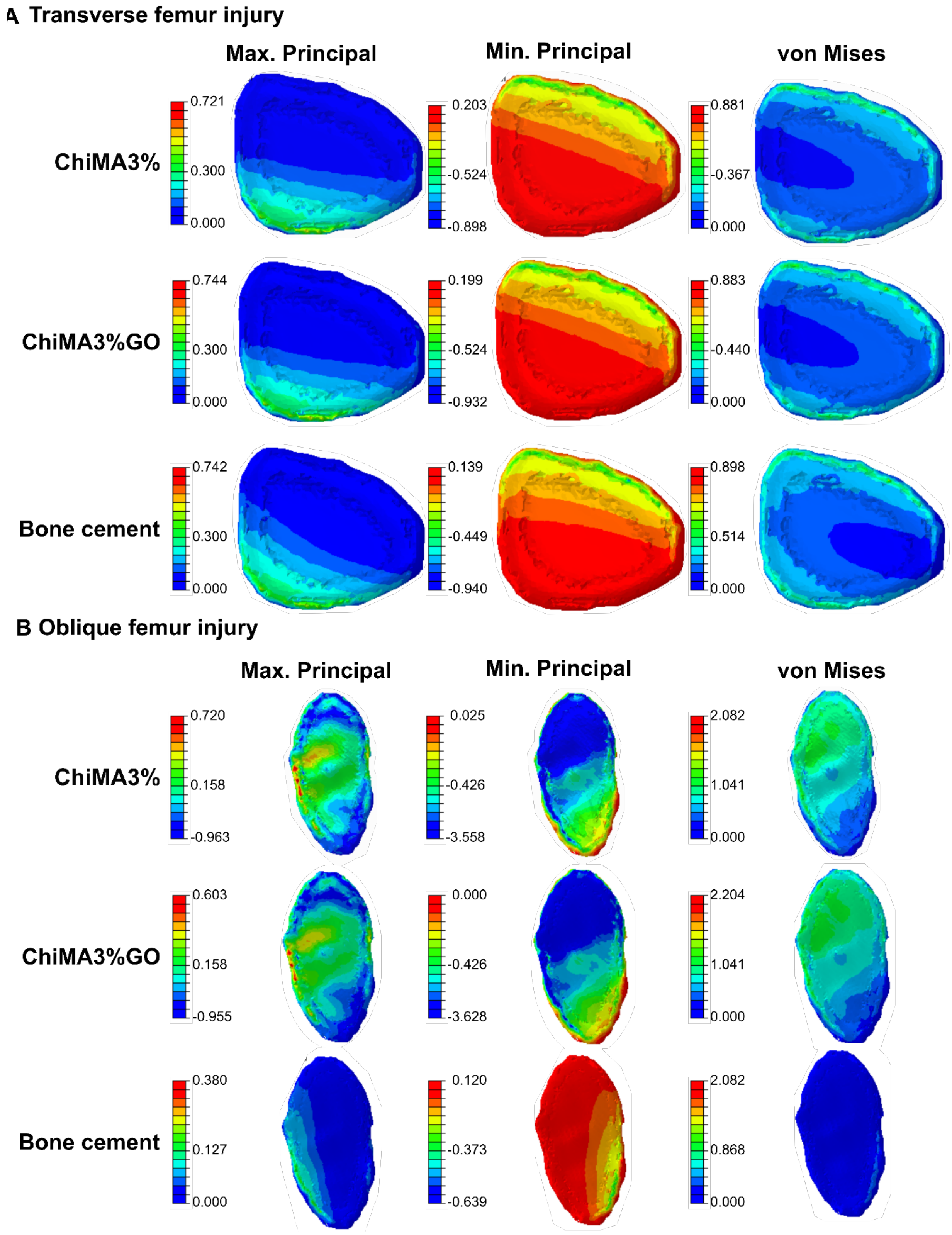

Figure S8. Distribution of tensile and compressive forces over ChiMA3%-, ChiMA3%GO- and bone cement-repaired femurs under the effect of hip momentum. Maximum principal, minimum principal, and von Mises stresses are shown for **(A)** the transverse lesion and **(B)** the oblique lesion.
